# Ocular Surface Disease: Advances in Diagnostics and Therapeutics

**DOI:** 10.1155/2018/6342130

**Published:** 2018-08-19

**Authors:** Zisis Gatzioufas, Samer Hamada, Sotiria Palioura

**Affiliations:** ^1^Department of Ophthalmology, University Hospital Basel, Basel, Switzerland; ^2^Corneo-Plastic Unit, Queen Victoria Hospital, East Grinstead, UK; ^3^Athens Vision Eye Institute, Athens, Greece; ^4^Bascom Palmer Eye Institute, University of Miami Miller School of Medicine, Miami, FL, USA

Ocular surface disease is an umbrella term that includes a variety of complex pathologies such as Stevens–Johnson syndrome, mucous membrane pemphigoid, limbal stem cell insufficiency, dry eye disease, and ocular graft-versus-host disease. Regardless of the underlying disease process, ocular surface failure may result in inflammatory and infectious complications and potentially devastating visual loss. Early diagnosis and appropriate treatment of ocular surface disease require a high level of expertise as these conditions can be extremely challenging even for experienced clinicians. Although key issues in the management of patients with complex ocular surface disease still remain controversial, recent advances in diagnostics and therapeutics have enhanced our armamentarium and allow for improved clinical outcomes.

In our special issue on ocular surface disease, Wróbel-Dudzińska et al. report on the clinical efficacy of platelet-rich plasma in the management of neurotrophic corneal ulcer, showing promising clinical results. Hazarbassanov et al. assess the effect of osmoprotection in the management of dry eye disease after refractive surgery in a randomized controlled double-blind clinical trial, while Moussa et al. investigate the effect of different prostaglandin analogues on the ocular surface of patients with primary open-angle glaucoma. Krysik et al. report on indications, outcomes, and complications of penetrating keratoplasty for ocular surface disease-related pathologies in a tertiary referral center, whereas Sun et al. analyze the therapeutic effects of corneal debridement combined with intrastromal voriconazole in a patient series with recalcitrant fungal keratitis. Ikegawa et al. investigate, with the aid of *in vivo* confocal microscopy, the morphology of two types of vortex keratopathy: amiodarone-induced keratopathy and the Fabry disease-associated keratopathy. Finally, Moschos et al. explore the psychological aspects and the incidence of depression in patients with symptomatic keratoconus.

*Zisis Gatzioufas* *Samer Hamada* *Sotiria Palioura*

## Conflicts of Interest

The authors declare that they have no conflicts of interest.

